# Supplementary material for: In-built thermo-mechanical cooperative feedback mechanism for self-propelled multimodal locomotion and electricity generation
Source: Nat Commun. 2018 Aug 24;9:3438. doi: 10.1038/s41467-018-06011-9 (PMC6109106; doi:10.1038/s41467-018-06011-9)
Supplement: Supplementary file 1 — Supplementary Information [file 41467_2018_6011_MOESM1_ESM.pdf]

Supplementary Information

In-built thermo-mechanical cooperative feedback mechanism for self-propelled multimodal locomotion and electricity generation

Wang et al.

Supplementary Figures

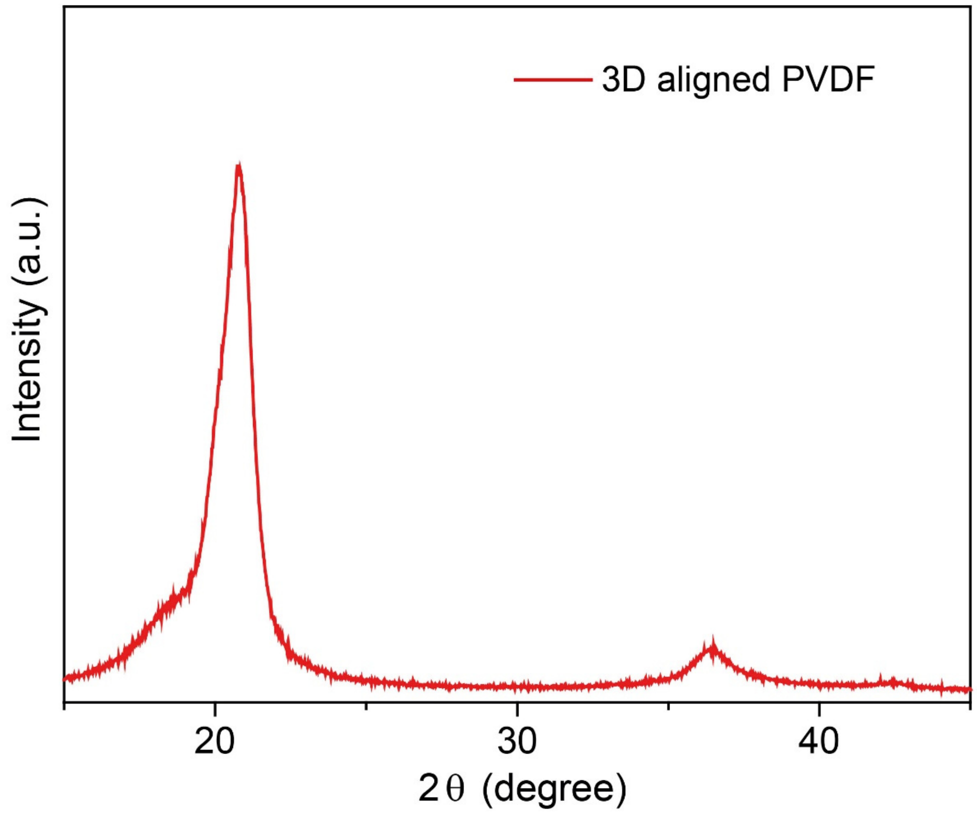

Supplementary Figure 1. XRD result of 3D aligned PVDF.

The β phase of PVDF has ferroelectric properties owing to a crystalline structure obtained by an all trans arrangement of the polymer chain. This gives rise to a permanent dipole[1], which makes it both a pyroelectric and piezoelectric nanogenerator.

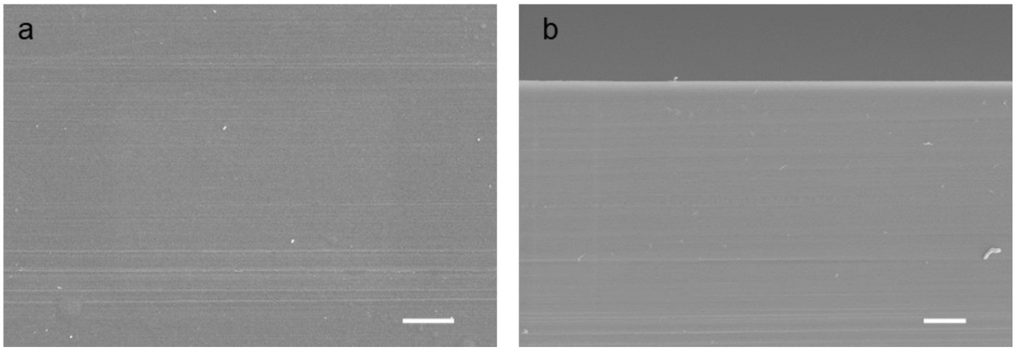

Supplementary Figure 2. (a) Surface and **(b)** cross-sectional scanning electron microscopy (SEM) image of 3D aligned PVDF film. The scale bar in (a) and (b) are 50 and 10 μm, respectively.

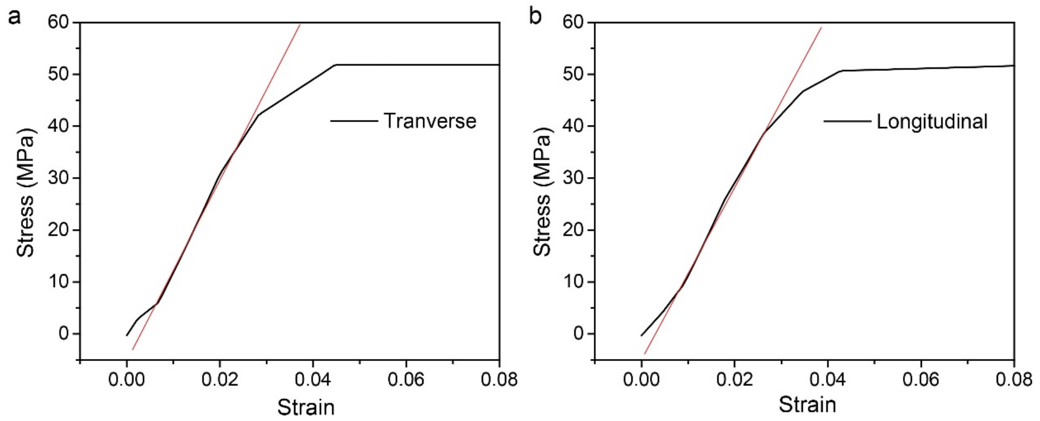

Supplementary Figure 3. Isotropic modulus of aligned PVDF. (a) Tensile strain stress curves of 3D aligned PVDF in the transverse and **(b)** longitudinal direction.

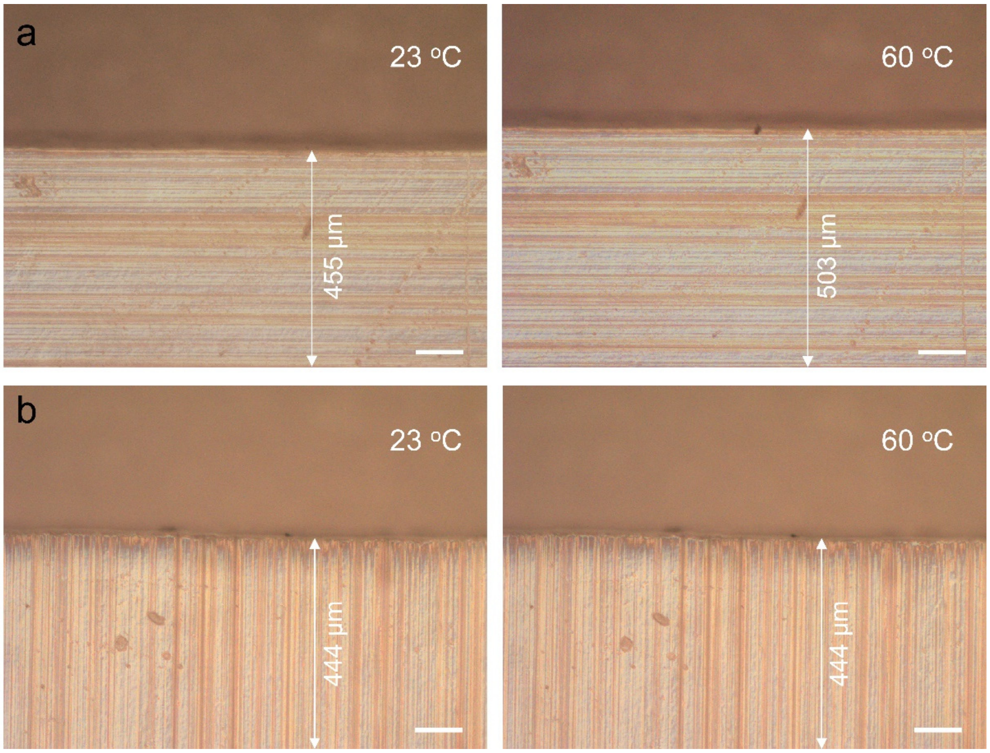

Supplementary Figure 4. Anisotropic CTE of aligned PVDF. (a) Optical micrographs of 3D aligned PVDF film with temperature increased from 23 to 60 °C in the transverse and **(b)** longitudinal direction. All scale bars correspond to 100 µm.

As the temperature was increased from 23 to 60 °C, the edge of the PVDF extended outward by 48 µm along the transverse direction, but not such an extension was founded in the longitudinal direction.

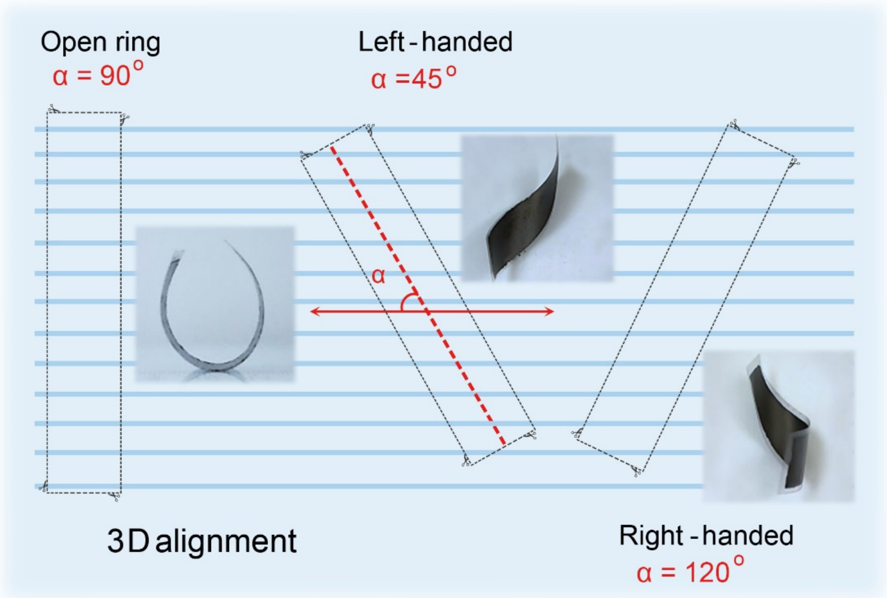

Supplementary Figure 5. Different thermo-mechanical deformation shapes of TMES strips depending on the alignment direction in which they are cut.

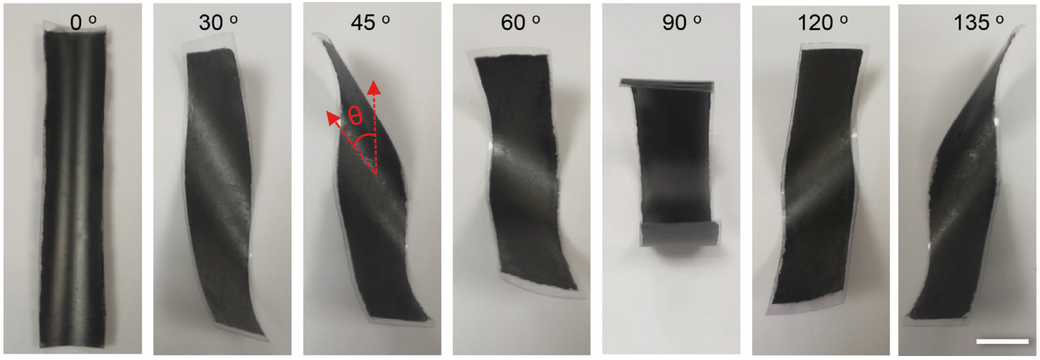

Supplementary Figure 6. Thermal actuated deformations of TMES at different alignment angles. The scale bar is 1 cm.

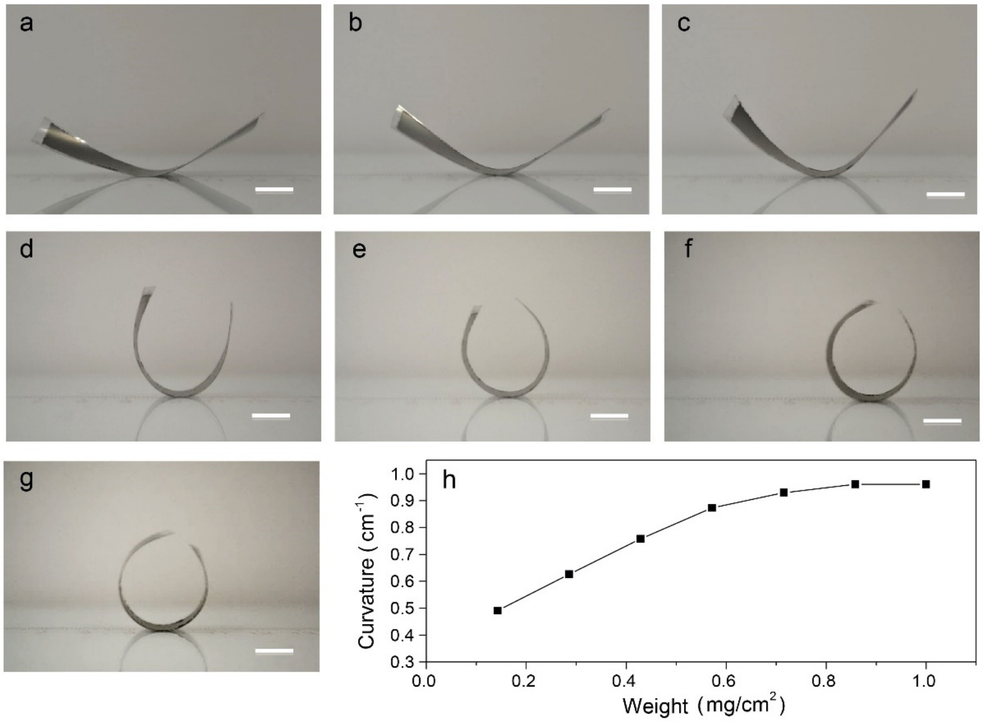

Supplementary Figure 7. Optimization of the thickness of PDG-CNT in TMES. Photograph and curvature of a TMES film with increased weight of PDG-CNT on a surface at 65 ^{o}C. When PDG-CNT on PVDF increases from 0.14 to 0.7 mg/cm^2, the curvature fast increases from 0.49 to 0.93; further increasing the weight of PDG-CNT result in little change in curvature. All scale bars correspond to 1 cm.

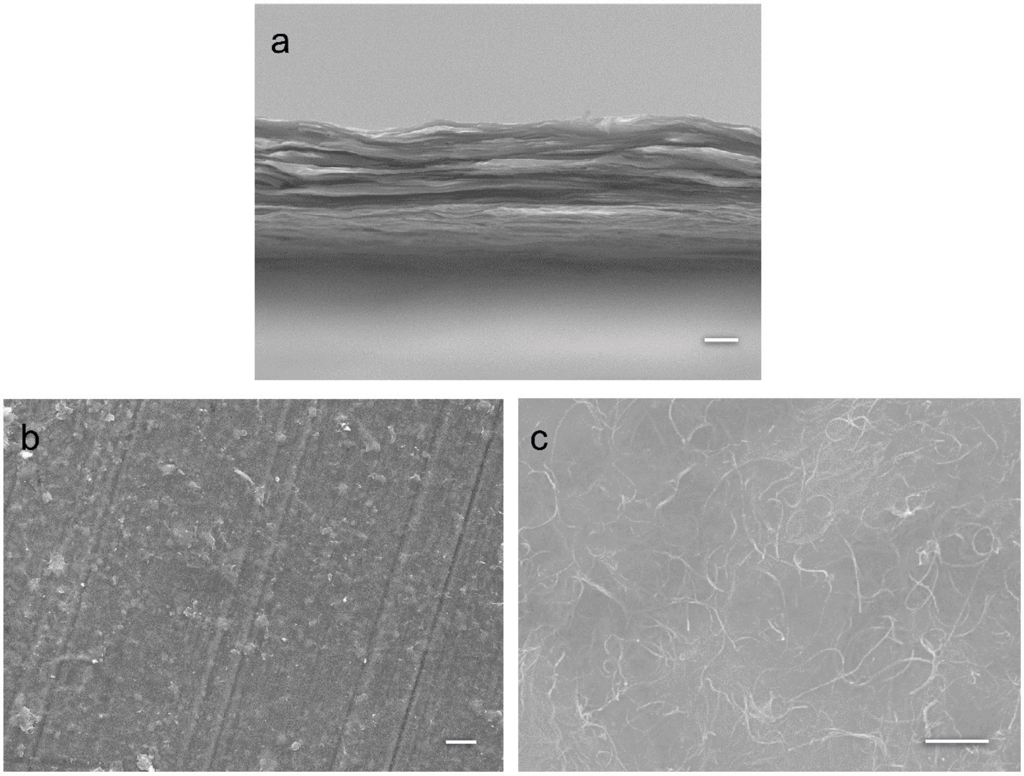

Supplementary Figure 8. SEM characterization of PDG-CNT. (a) SEM image of cross-section, **(b)** surface and **(c)** amplified surface of PDG-CNT layer in TMES. The scale in (a), (b) and (c) are 2 μm, 10 μm and 500 nm, respectively.

The thickness of PDG-CNT on PVDF (0.8 mg/cm^2) was measured to be 5.0 μm (Supplementary Fig. 8a). As a result, at a fixed thickness of PVDF (~ 80 μm), the thickness of PDG-CNT is optimized at 5.0 μm for a maximized thermo-mechanical deformation behavior. The 3D aligned PVDF and PDG-CNT are tightly bonded together, and we can even observe the lamellar orientation on the surface of PDG-CNT layer in TMES (Supplementary Fig. 8b,c).

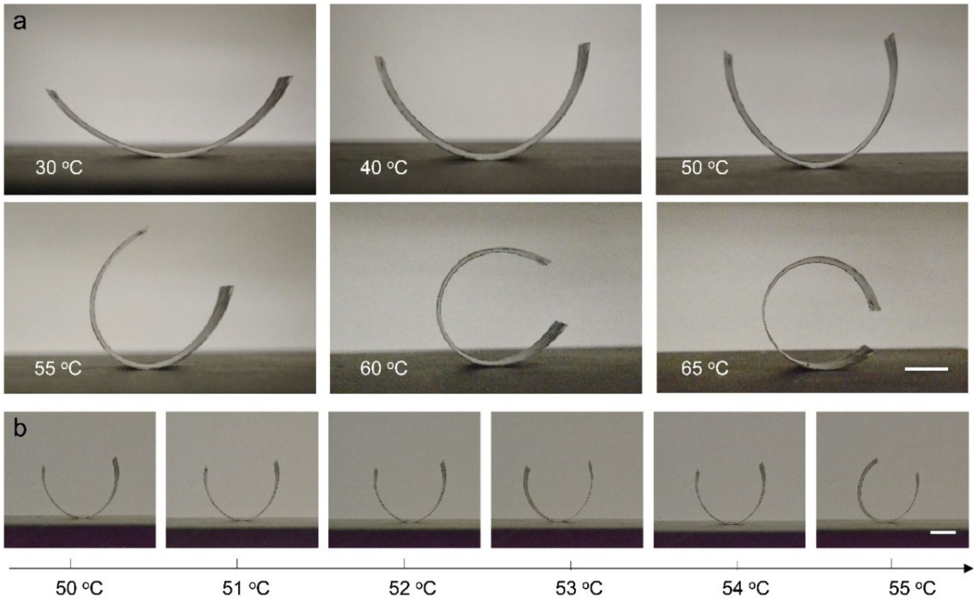

Supplementary Figure 9. Thermal actuated deformations of the TMES strip at α = 90 °. **(a)** Photograph of TMES on a hot surface with temperature increased from 30 to 65 °C. **(b)** Photograph showing slight swinging of TMES to stable oscillation with hot surface temperature one degree by one degree increased from 50 to 55 °C. All scale bars correspond to 1 cm.

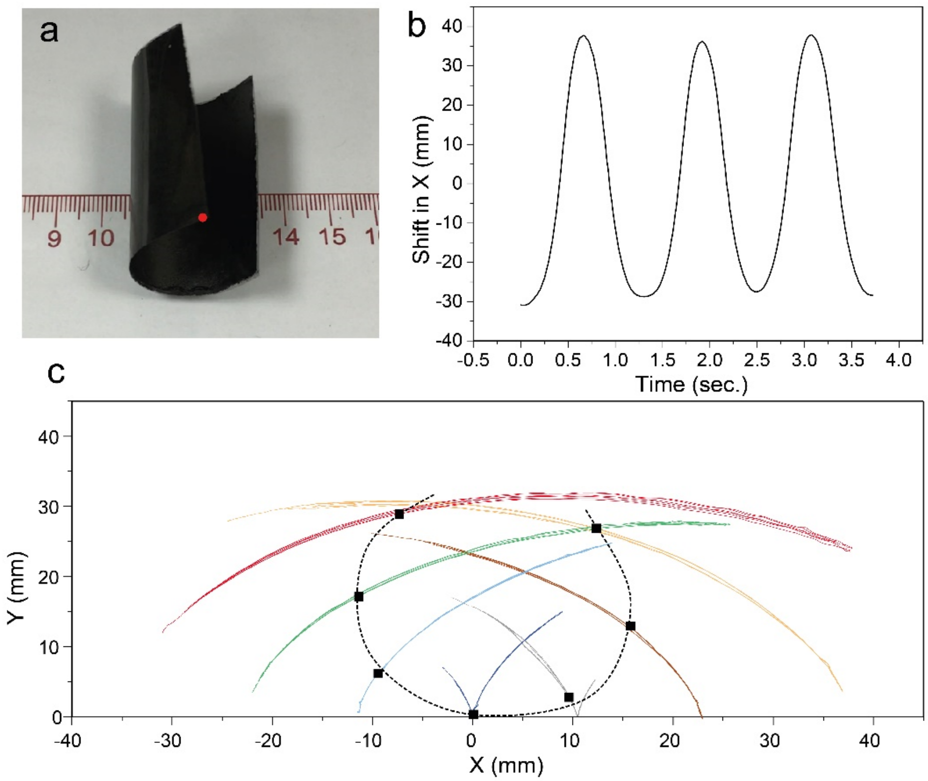

Supplementary Figure 10. Thermal actuated oscillation behavior of a square-shaped TMES at α = 90 °. (a) Photograph of TMES with size of 6 × 6 cm^2 on hot surface at 60 °C. **(b)** The 2D trajectory of tracked points on TMES in three consecutive oscillation cycles. **(c)** The shift of point 1 in the *x*-axis in three consecutive oscillation cycles.

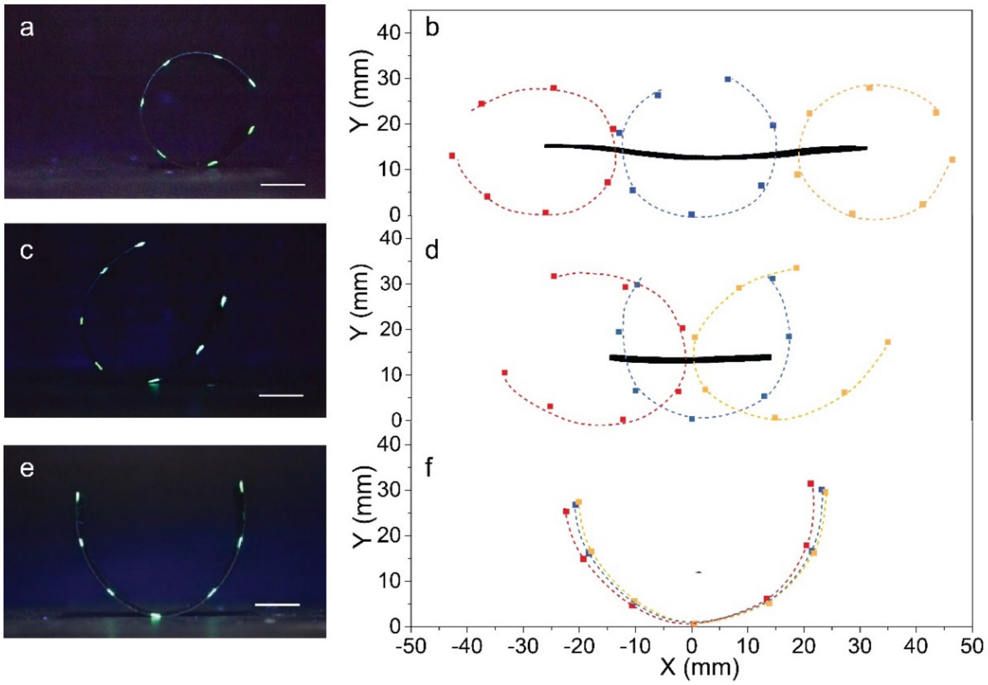

Supplementary Figure 11. Centroid trajectory tracking during the oscillation process.

(a, b) Fluorescent image of tracked points on TMES and centroid trajectory on hot surface at 65 °C, **(c, d)** 55 °C and **(e, f)** 50 °C. All scale bars correspond to 1 cm.

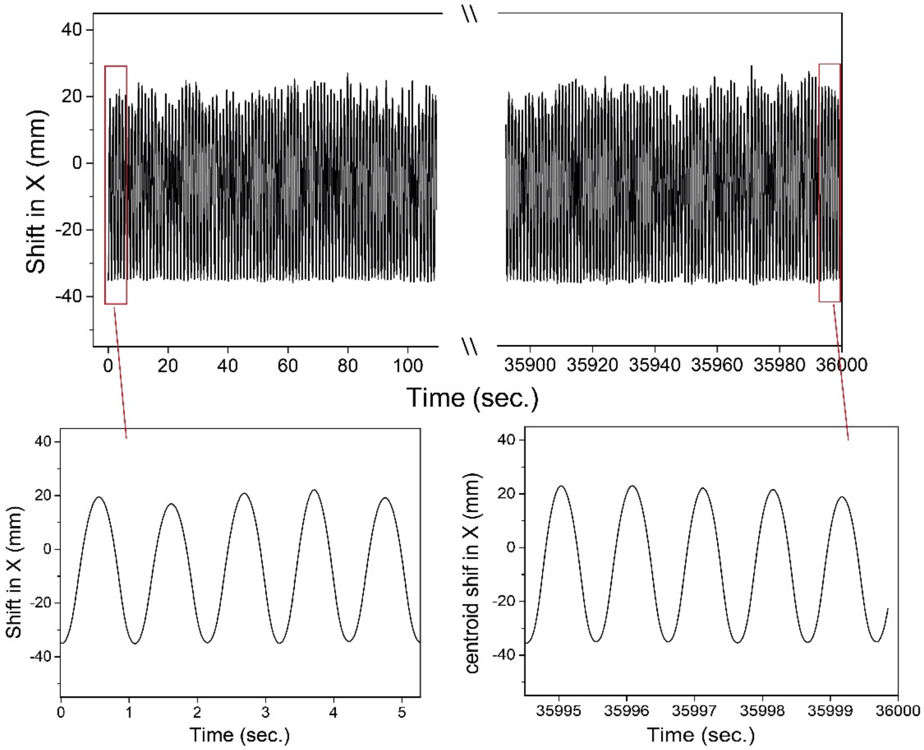

Supplementary Figure 12. Stability test of oscillation behavior. Shift of the leftmost point on TMES in the *x*-axis over 10 hours of oscillations on the hot surface at 55 ^{o}C.

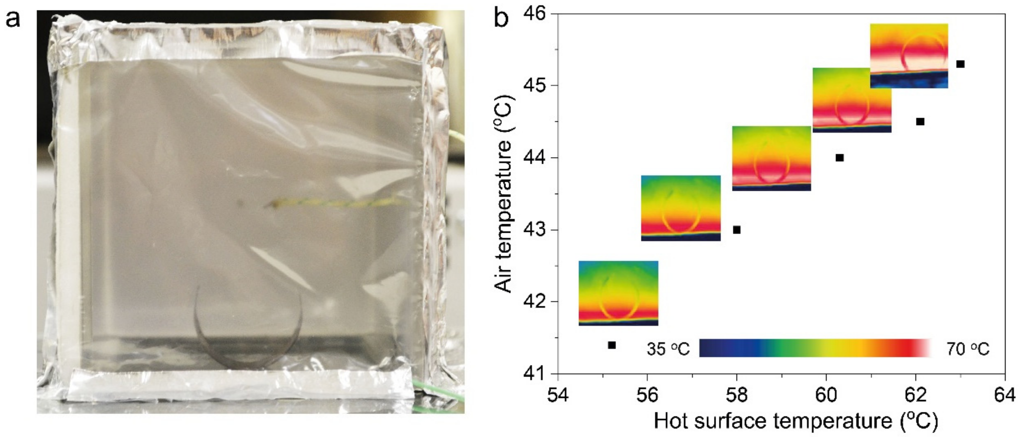

Supplementary Figure 13. Contrast experiments of thermo-mechanical behavior of TMES in closed space. (a) The set up for measuring the response of TMES on hot surface in a closed box. **(b)** The response of TMES to different combinations of air and hot surface temperatures.

As a demonstration test, we found that the oscillation could not be activated even when large deformations were produced on the hot surface in a closed box due to the high air temperatures.

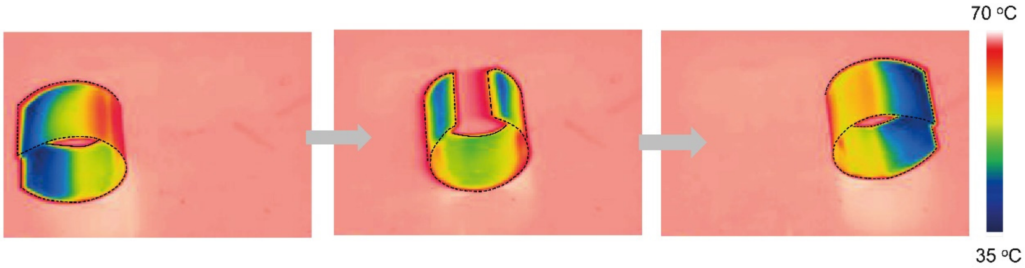

Supplementary Figure 14. Infrared images of TMES during the oscillation process.

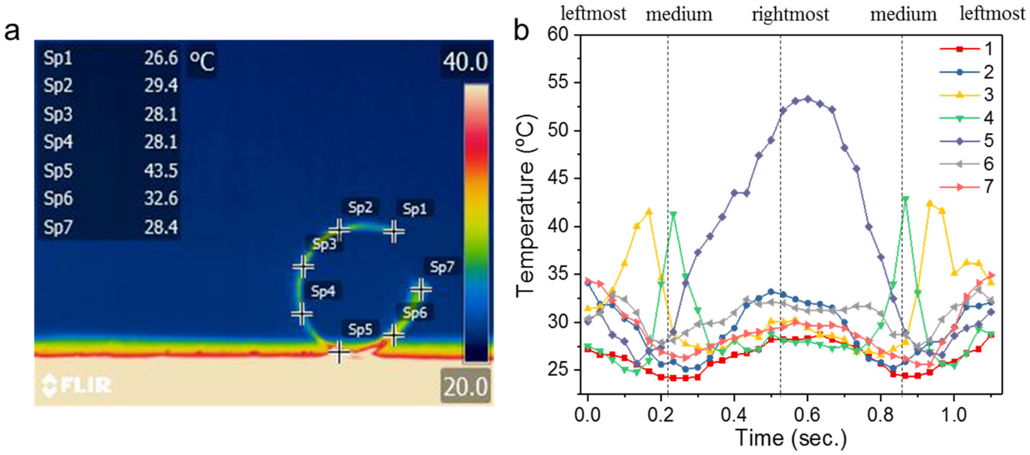

Supplementary Figure 15. Temperature profile of marked TMES. (a) An infrared image of TMES with marked points on the hot surface at 60 °C. **(b)** Temperature profile of the marked points in one oscillation cycle.

The points near the extreme ends, 1, 2, 6 and 7, undergo thermal fluctuations in a small temperature range of 25 to 35 °C, and the highest temperature of point 3 and 4 reaches 42 °C when they contact the hot surface. The temperature of point 5 can rise to as high as 54 °C due to its relative long duration in proximity to/contact on the hot surface during the period when TMES oscillates from the medium to rightmost and then back to medium position (~ 0.6 s).

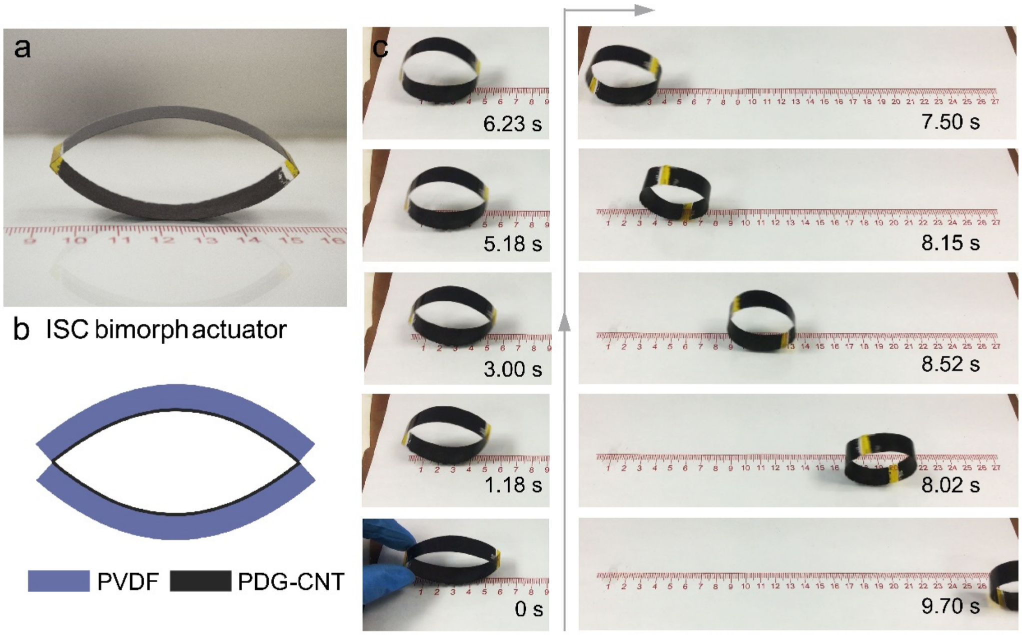

Supplementary Figure 16. Self-rolling behavior of an ISC bimorph actuator.

(a) Photograph of an ISC bimorph actuator. **(b)** Schematic of configuration of the ISC bimorph actuator. **(c)** Self-rolling forward motion of the ISC bimorph actuator on hot surface at 80 ^{o}C.

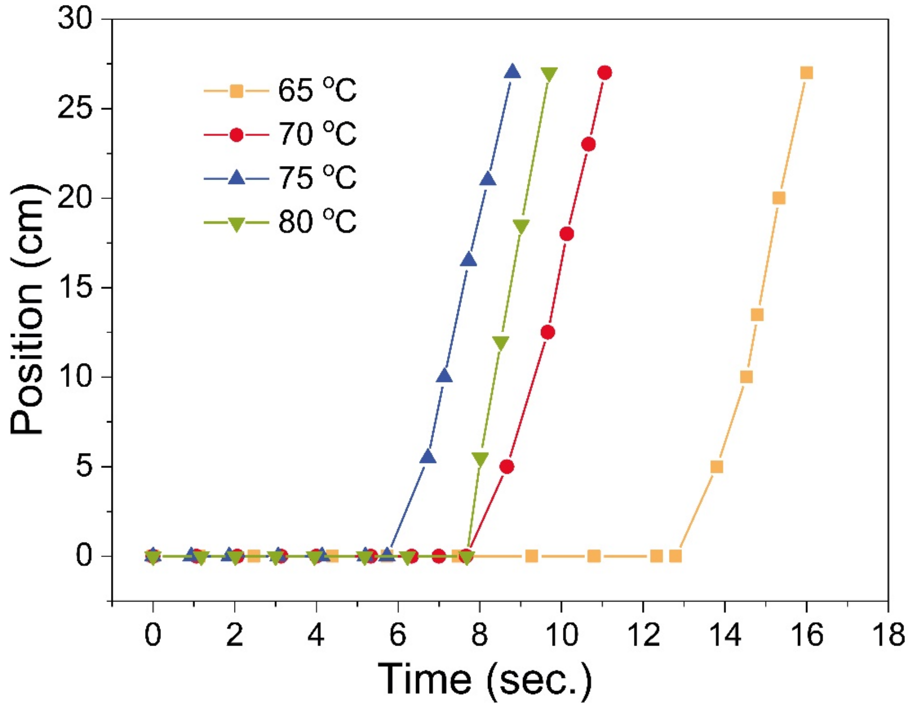

Supplementary Figure 17. Self-rolling forward motion of the ISC bimorph actuator at different temperatures. The position of ISC actuator depending on time after placed on a hot surface at 65, 70, 75 and 80 °C, respectively.

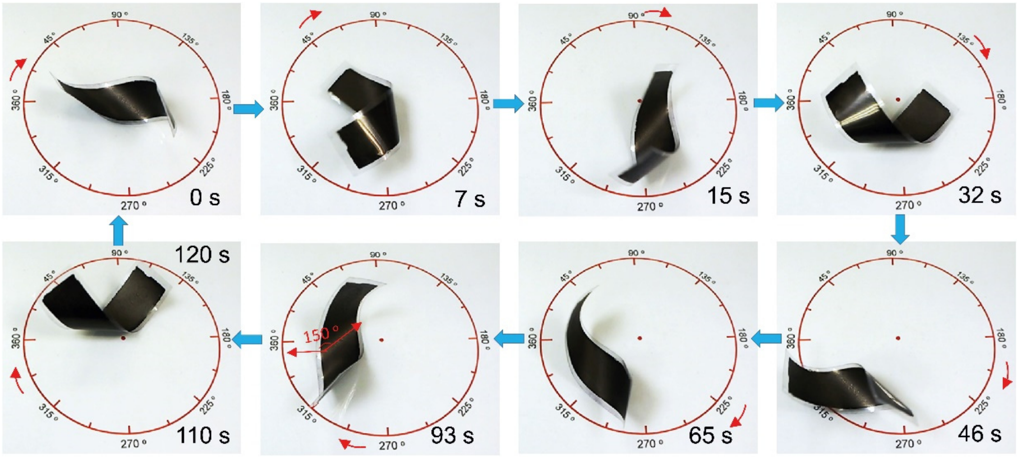

Supplementary Figure 18. Clockwise rotation of the right-handed twisted TMES at α = 120 º.

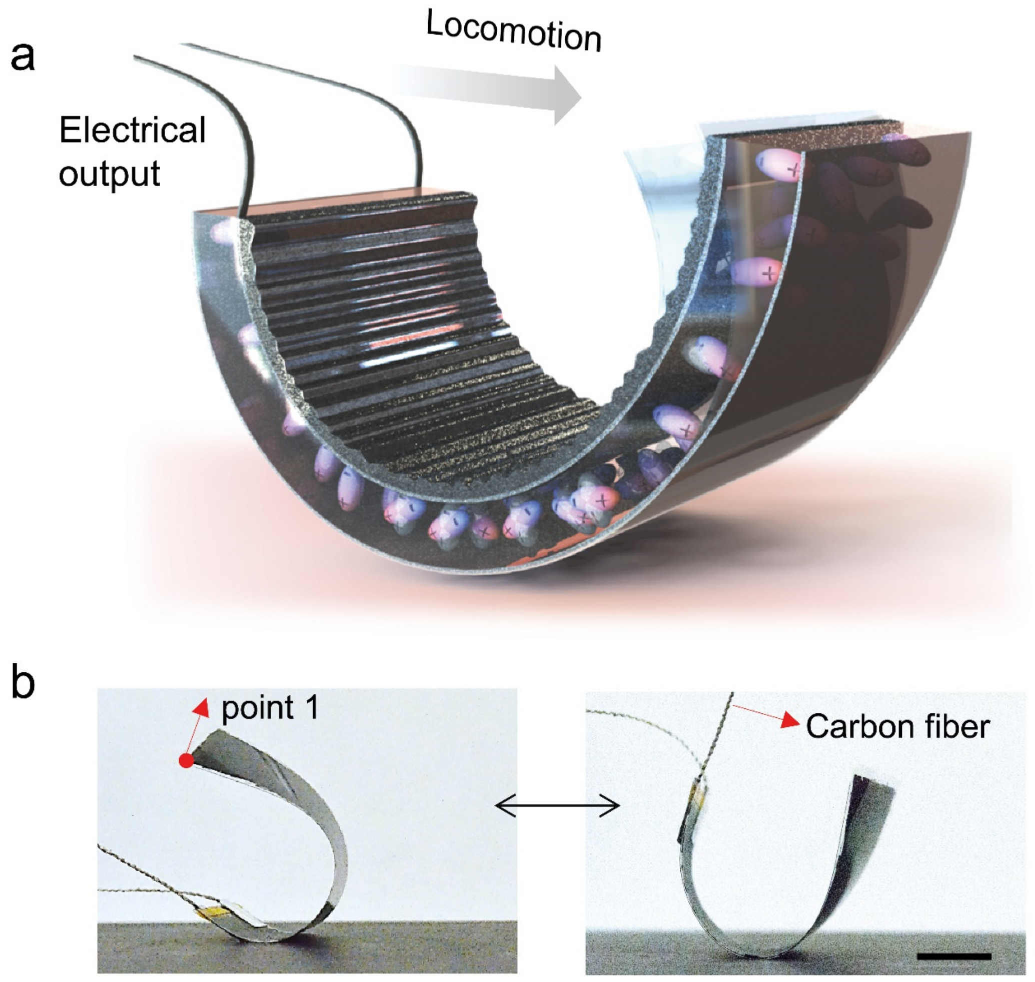

Supplementary Figure 19. The device of TMES for harvesting of thermomechanical locomotion energy. (a) Schematic illustration of the thermo-mechanical locomotion energy harvesting. **(b)** Photograph of TMES tethered with carbon fiber electrodes during the energy harvesting process. The scale bar is 2 cm.

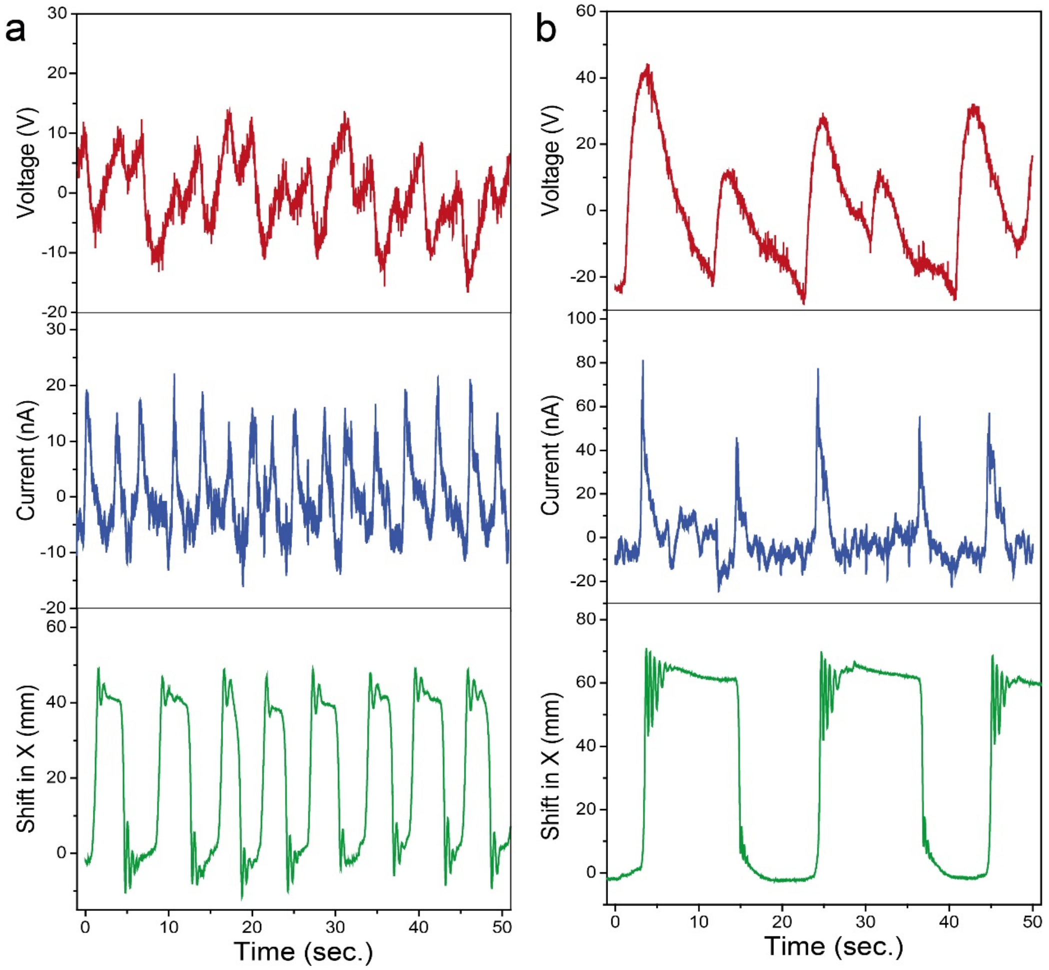

Supplementary Figure 20. Pyro/piezoelectricity generation of TMES strips during the thermo-mechanical locomotion process. (a) The oscillation trajectory in the *x*-axis, output short circuit current and open circuit voltage on hot surface at 55 ^{o}C and **(b)** 65 ^{o}C.

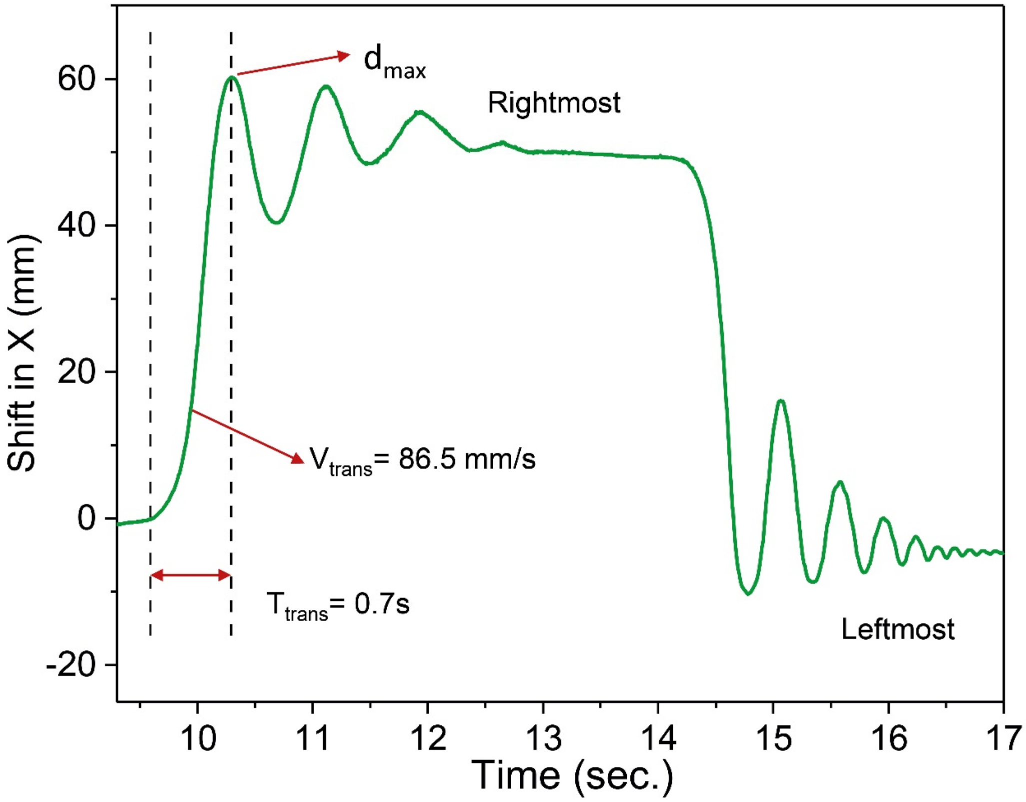

Supplementary Figure 21. The maximum displacement and transition speed of TMES generator.

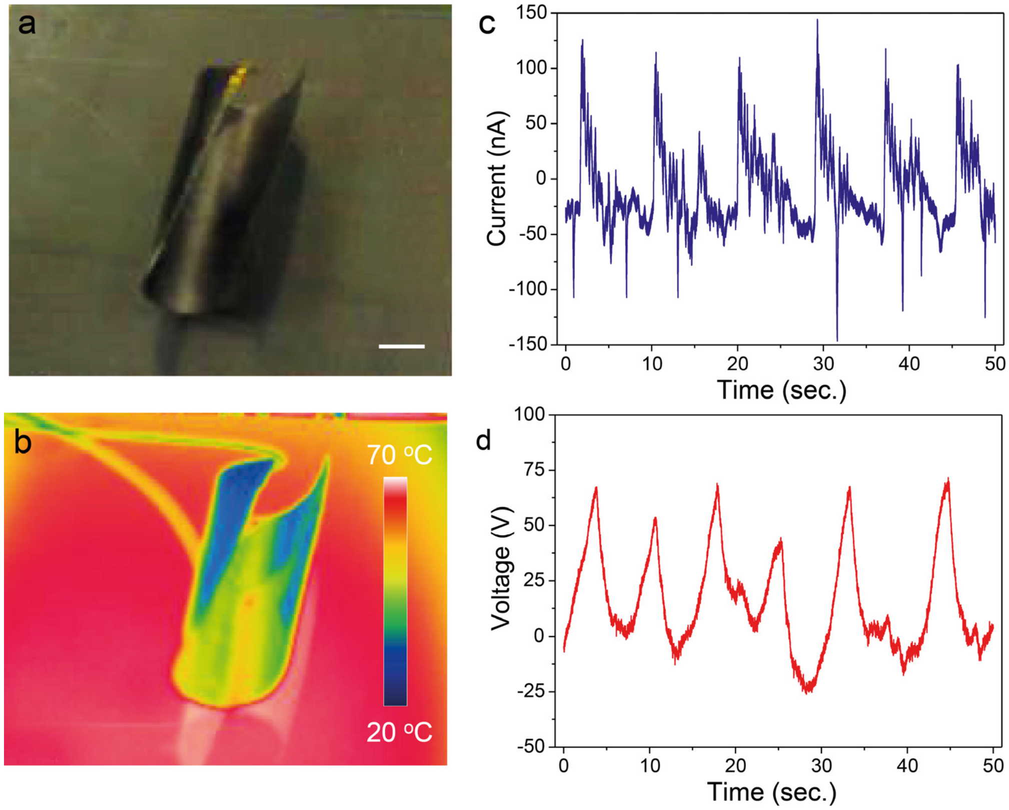

Supplementary Figure 22. Electricity generation performance of square-shaped TMES.

(a) Photograph and **(b)** infrared image of TMES with size of 6 × 6 cm^2 on hot surface at 60 °C. **(c)** Output short circuit current and **(d)** open circuit voltage within 50 s. The scale bar is 1 cm.

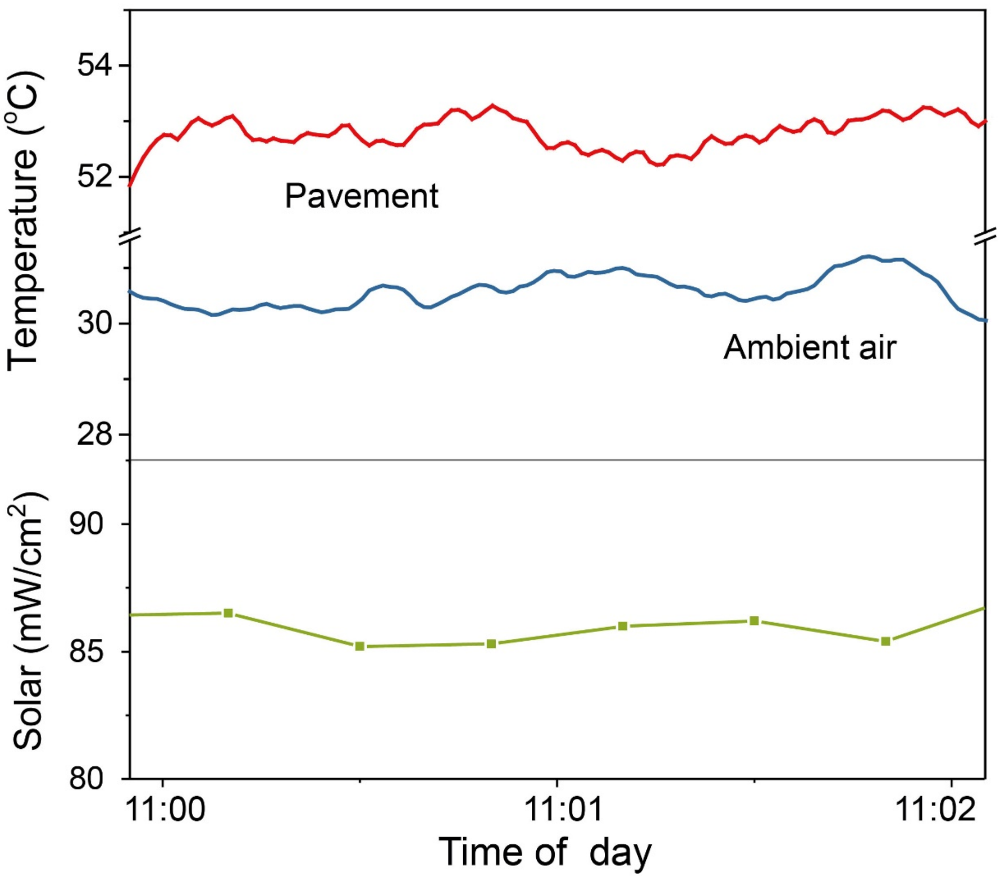

Supplementary Figure 23. Time-dependent solar intensity, temperature of ambient air and the surface of a black tiled pavement.

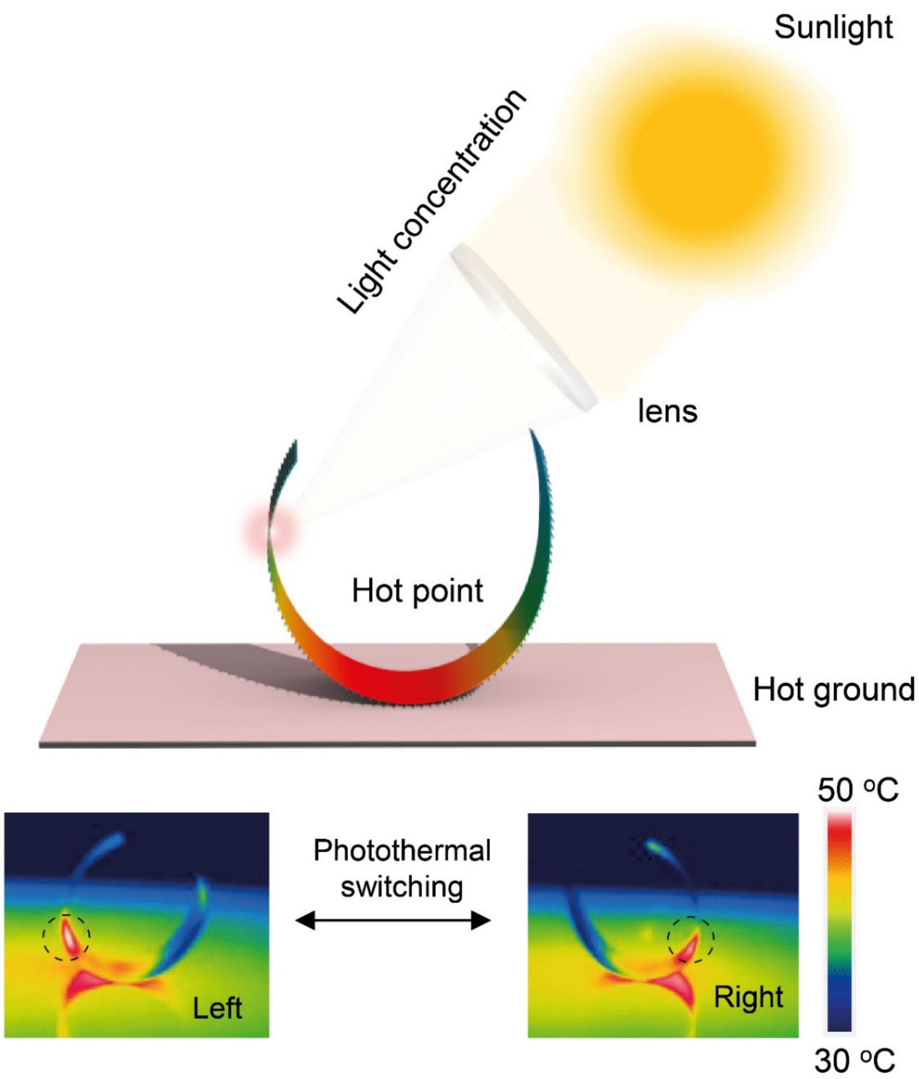

Supplementary Figure 24. Schematic illustration and infrared images of sunlight directed mechanical work of TMES.

Supplementary Note

Supplementary Note 1

Calculated energy conversion efficiencies of TMES

With regards to the energy conversion efficiency, we postulate that the energy conversion to be as such:
Waste heat energy → Gain in gravitational potential energy (due to deformation and raising of centroid) → Kinetic energy and electrical energy
Energy conversion efficiency η is,

$$\frac{\text{Gain in gravitational potential energy}}{\text{Total heat input}}$$

To estimate the total heat input, we assume the hot surface is an ideal blackbody of 60 °C, having surface area of 1.5 cm by 6 cm. Using Stefan-Boltzmann law, the power provided by the hot surface is 0.6287 W or 0.6916 J in one oscillatory cycle of 1.1 s.
The potential energy of the system is mgh, approximately 2.5×10^{-5} J, where the centroid of TMES is raised by 12.5 mm and the weight is 0.2 g.
The efficiency of energy conversion is approximately 0.004 %.

The conversion of energy from gravitational potential energy to kinetic energy and electrical energy is more efficient. The kinetic energy consists of both the translational and rotational kinetic energy of TMES. The translational kinetic energy $\left(\frac{1}{2}mv^2\right)$, computed from the lateral speed of the centroid of TMES (88.7mm/s) is 7.868×10^{-7} J. The rotational kinetic energy $\left(\frac{1}{2}I\omega^2\right)$ is computed as shown below.
Assuming that TMES is an incomplete thin wall circular cylinder,

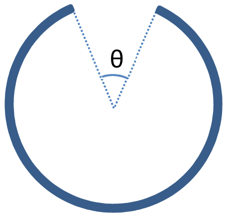

Radius of curvature: 11.1 mm
$I = mR^2$
$\rightarrow I \approx 24.642$ g.mm^2
$\omega = \frac{v}{r} \approx 8$ rad/s
Rotational kinetic energy $\approx 1.577 \times 10^{-6}$ J

Taking the integral of the voltage and current ($\int_{t_0}^{t_1} V.Idt$), where t_0 and t_1 are start and end time of one oscillatory cycle respectively, the total electrical energy generated is 6.94×10^{-7}J. As a result, the efficiency of conversion from potential energy to kinetic and electrical energy is approximately 12.2 %.

Supplementary Reference

1. Bowen, C. R. *et al.* Pyroelectric materials and devices for energy harvesting applications. *Energy Environ. Sci.* **7**, 3836-3856 (2014).
