# Supplementary material for: In-built thermo-mechanical cooperative feedback mechanism for self-propelled multimodal locomotion and electricity generation
Source: Nat Commun. 2018 Aug 24;9:3438. doi: 10.1038/s41467-018-06011-9 (PMC6109106; doi:10.1038/s41467-018-06011-9)
Supplement: Supplementary file 3 — Description of Additional Supplementary Files [file 41467_2018_6011_MOESM3_ESM.docx]

**Description of Additional Supplementary Files**

**Supplementary Movie 1.** Self-oscillation of a TMES film (*α* = 90 ^o^) on a hot surface at 55 ^o^C.

**Supplementary Movie 2.** Fluorescence labeling and tracking of one oscillation cycle.

**Supplementary Movie 3.** Oscillation stability tested for 1200 times on a hot surface at 55 ^o^C.

**Supplementary Movie 4.** Dynamic thermal analysis for the oscillation process on a hot surface at 60 ^o^C.

**Supplementary Movie 5.** Self-rolling forward motion of a TMES film (*α* = 90 ^o^) on a hot surface at 70 ^o^C and ISC bimorph actuator at 80 ^o^C.

**Supplementary Movie 6.** Anticlockwise rotation of a TMES film (*α* = 45 ^o^) on a hot surface at 70 ^o^C.

**Supplementary Movie 7.** Clockwise rotation of a TMES film (*α* = 120 ^o^) on a hot surface at 70 ^o^C.

**Supplementary Movie 8.** Grasping a sponge and performing oscillation work of TMES film (*α* = 90 ^o^) on a hot surface at 65 ^o^C, cargo transportation across a hot surface at 70 ^o^C and see-saw balance work driven by hot surface at 60 ^o^C.

**Supplementary Movie 9.** Dynamic thermal analysis for the thermo-mechanical energy harvesting for pyro/piezoelectricity output process.

**Supplementary Movie 10.** Thermo-mechanical oscillation energy harvesting process and synchronous Isc output.

**Supplementary Movie 11.** Self-defensive motions of a prototypical TMES-bot on a hot surface at 70 ^o^C.

**Supplementary Movie 12.** Autonomous locomotion of a TMES film (*α* = 90 ^o^) driven by solar heat under an ambient outdoor environment.
